# Genome-wide microarray analysis of Atlantic cod (*Gadus morhua*) oocyte and embryo

**DOI:** 10.1186/1471-2164-15-594

**Published:** 2014-07-14

**Authors:** Adrijana Škugor, Aleksei Krasnov, Øivind Andersen

**Affiliations:** Nofima, Osloveien 1, N-1432 Ås, Norway; Department of Animal and Aquaculture Sciences, Norwegian University of Life Sciences, N-1432 Ås, Norway

**Keywords:** Atlantic cod, Oocyte, Embryo, Development, Microarray

## Abstract

**Background:**

Regulation of gene expression plays a central role in embryonic development. Early stages are controlled by gametic transcripts, which are subsequently substituted with transcripts from the genome of the zygote. Transcriptomic analyses provide an efficient approach to explore the temporal gene expression profiles in embryos and to search for the developmental regulators. We report a study of early Atlantic cod development that used a genome-wide oligonucleotide microarray to examine the composition and putative roles of polyadenylated transcripts.

**Results:**

The analyses were carried out in unfertilized oocytes, newly fertilized oocytes and embryos at the stages of mid-blastula transition and segmentation. Numerous genes transcribed in oocytes are involved in multiple aspects of cell maintenance and protection, including metabolism, signal perception and transduction, RNA processing, cell cycle, defense against pathogens and DNA damage. Transcripts found in unfertilized oocytes also encoded a large number of proteins implicated in cell adherence, tight junction and focal adhesion, suggesting high complexity in terms of structure and cellular interactions in embryos prior to midblastula transition (MBT). Prezygotic transcripts included multiple regulators that are most likely involved in developmental processes that take place long after fertilization, such as components of ErbB, hedgehog, notch, retinoid, TGFb, VEGF and Wnt signaling pathways, as well as transcripts involved in the development of nervous system. The major event of MBT was the activation of a large group of histones and other genes that modify chromatin structure preceding massive gene expression changes. A hallmark of events observed during segmentation was the induction of multiple transcription factors, including a large group of homeobox proteins in pace with decay of a large fraction of maternal transcripts. Microarray analyses detected a suite of master developmental regulators that control differentiation and maintenance of diverse cell lineages.

**Conclusions:**

Transcriptome profiling of the early stages in Atlantic cod revealed the presence of transcripts involved in patterning and development of tissues and organs long before activation of the zygotic genome. The switch from maternal to zygotic developmental programs is associated with large-scale modification of chromosomes.

**Electronic supplementary material:**

The online version of this article (doi:10.1186/1471-2164-15-594) contains supplementary material, which is available to authorized users.

## Background

Early ontogeny is associated with dramatic gene expression changes that underlie and determine the developmental processes. Transcription terminates by the end of oogenesis when the maturing oocyte is arrested in the metaphase of its second meiotic division [1, 2]. The oocyte is loaded with maternal mRNAs and proteins that control the cell maintenance and fate and the formation of the body plan prior to the onset of zygotic genome expression [3, 4]. Important transcripts can be also contributed by sperm cell, as was recently shown in *Drosophila* and mammals [5, 6]. Today, it is generally thought that the combination of determinants deposited by the mother during oogenesis and the inductive signals between different cells trigger the specification of different cell lineages during development of the embryo [7, 8]. Maternal to zygotic transition (MZT) is the key event during embryogenesis marked by the switch of control from the maternal and possibly paternal transcripts to the newly synthesized embryonic gene products [9–11]. Degradation of maternal transcripts and zygotic genome activation is characterized by striking changes in the transcriptome profiles. MZT timing is species-specific according to the extent and form of maternal contributions and generally occurs earlier in mammals [12–15] compared to fish, *Drosophila* and *Xenopus* [16–19]. In a number of animal species, MZT roughly coincides with the mid-blastula transition (MBT) [20] when cells become motile and divide asynchronously. The three germ layers and the body plan of the mature organism are established during gastrulation, and the period is characterized by extensive cell movements and intracellular communications [21, 22]. During the following segmentation stage major events in the formation of tissues and organs take place.

Knowledge of the genetic networks controlling embryogenesis has been obtained principally by mutagenesis screens in model species. Multiple mutations affecting embryonic development have been induced by chemical and insertional mutagenesis resulting in the identification of genes with important roles in development in *Drosophila* [23–25]. Similarly, large-scale genetic screens in zebrafish have enhanced the overall understanding of critical steps and pathways during embryogenesis, and forward genetics revealed a number of developmentally regulated genes [26–28]. Despite high power, this research strategy encounters limitations because only indispensable genes whose loss cannot be compensated by functionally related genes are found, leaving many important actors undetected. A complementary approach is transcriptome profiling that reveals genes with characteristic temporal expression patterns. The completion of the Atlantic cod whole-genome sequencing project [29] enabled the development of novel tools for gene expression profiling of this ecologically and commercially important marine species sustaining wild fisheries and aquaculture. DNA microarrays are used for analyses of polyadenylated mRNA and a transcriptome study of Atlantic cod embryogenesis using a cDNA microarray was recently reported [30]. We present herein the use of the Atlantic cod genome-wide oligonucleotide microarray for investigation of transcriptome changes associated with the key events of early development from unfertilized oocytes to late somitogenesis with focus on changes during MZT. Contribution of transcripts with different temporal profiles in diverse processes associated with maintenance and development was assessed and compared.

## Results

### An overview of oocyte and embryo transcriptome

**Figure 1:**
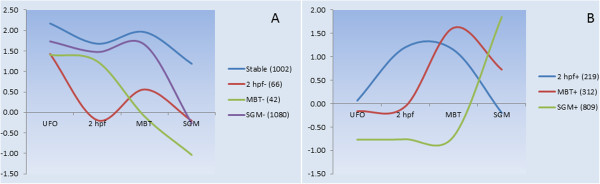
**Developmental profiles of polyadenylated mRNA.** Transcripts were categorized as prezygotic **(A)** or zygotic **(B)** and divided into subgroups as explained in Methods. Data are mean log_2_-expression ratio, the numbers of features are in parentheses.

The microarray analyses of polyadenylated mRNA included four developmental stages: unfertilized oocytes (UFO), oocytes collected at 2 hours post fertilization (2hpf), the midblastula transition (MBT) and segmentation (SGM). The features that showed over 2-fold difference in comparison with reference (adult tissues) in at least one of the analysed stages were selected. These genes were categorized as prezygotic (high expression in UFO) and zygotic (activation after fertilization), and further divided in seven subgroups based on the decreased or increased abundance at the specific stages (Figure 1, Table 1, Additional file 1). Most prezygotic transcripts maintained relatively stable levels either within the whole period examined or until SGM. A small number of transcripts decreased or increased abundance shortly after fertilization. The origin of the transcripts detected at the 2 hpf is uncertain, but the zygote formation in Atlantic cod occurs approximately at 5 hpf [31]. Therefore, most if not all these transcripts identified at 2 hpf most likely should be ascribed to the sperm cell. Genes activated at MBT and SGM comprised respectively 17.2% and 80.4% of all zygotic genes.

**Table 1:** **Division of genes in groups by temporal expression profiles**

Groups	Criteria
***Prezygotic***	log2-ER > 0.8 in UFO
Prezygotic 2 hpf-	log2-ER < 0.3 in 2 hpf
Prezygotic MBT-	log2-ER < 0.3 in MBT
Prezygotic SGM-	log2-ER < 0.3 in SGM
Prezygotic stable	The rest prezygotic
***Zygotic***	log2-ER < 0.8 in UFO
Zygotic 2 hpf+	log2-ER > 0.8 in 2 hpf
Zygotic MBT+	log2-ER > 0.8 in MBT
Zygotic SGM+	log2-ER > 0.8 in SGM

### Metabolism, cell maintenance, proliferation and protection

**Figure 2:**
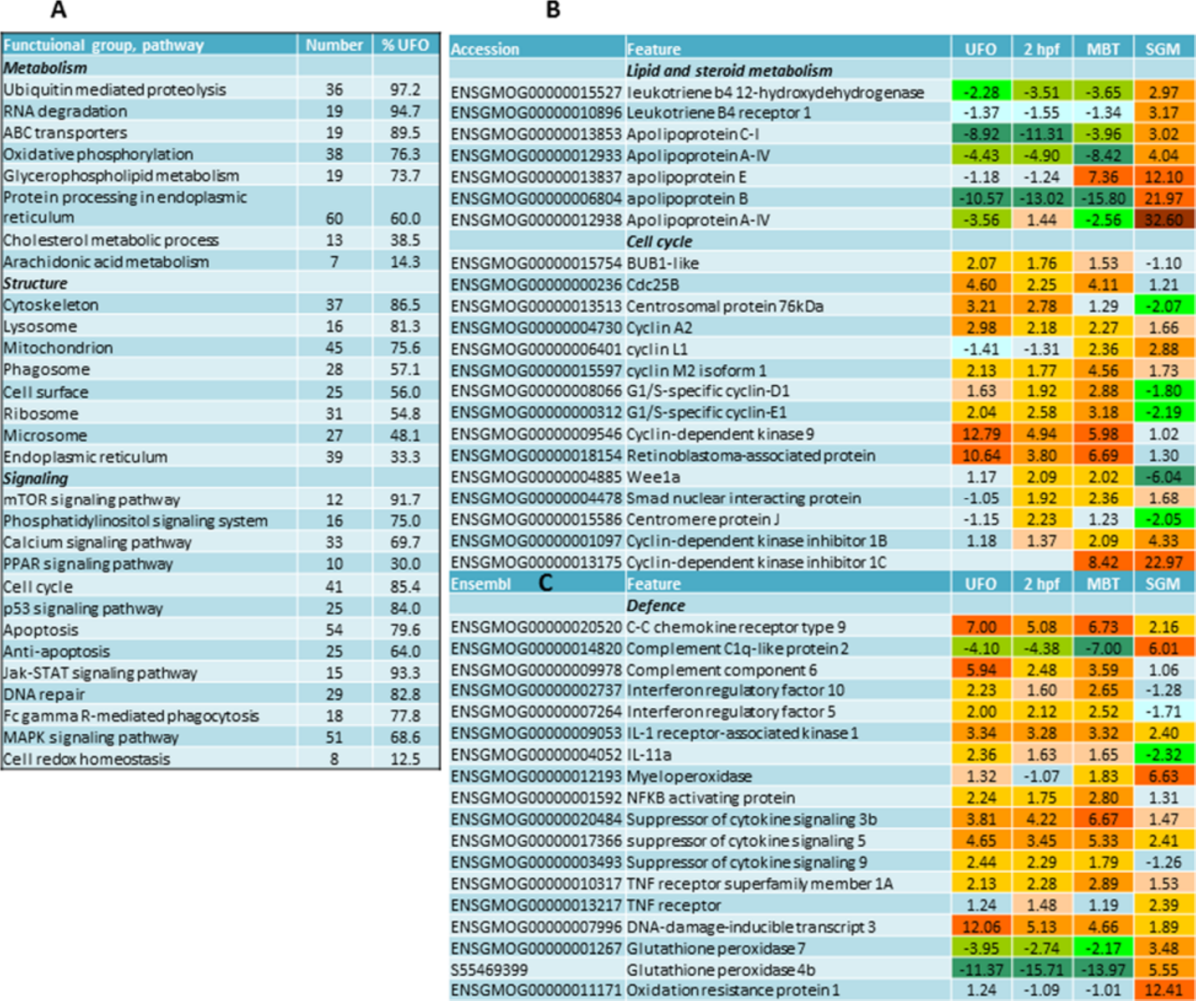
**Metabolism, cell maintenance, proliferation and protection. A**: numbers of genes by functional classes of GO and pathways of KEGG. **B**: expression of genes involved in lipid and steroid metabolism and cell cycle regulation. **C**: expression of genes involved in defense against pathogens. Data are fold ratios to reference (adult tissues) in this and the following heat maps.

Genes with metabolic roles comprised a large part of the developmentally regulated genes. Functional groups involved in degradation of proteins and RNA were represented almost exclusively by prezygotic transcripts that suggested a profound reconstruction of the cellular machinery (Figure 2A). This was in line with zygotic activation of multiple genes encoding components of the key cellular organelles and structures: cell surface, ribosomes and microsomes with the greatest changes observed in the endoplasmic reticulum. Proteins of cytoskeleton, mitochondria and lysosomes were predominantly dependent on maternal transcripts, as well as signalling pathways controlling metabolic processes. The greatest developmental regulation was seen in the pathways of lipid and cholesterol metabolism and PPAR signalling; a sharp induction at SGM was shown by a suite of apolipoproteins (Figure 2B). Proteins involved in cell cycle and apoptosis were encoded predominantly by the maternal transcripts. *Cdc25B*, which induces mitosis by promoting G2/M phase progression, was abundant during the early stages followed by gradual decreasing levels towards the SGM. Consistently, the elimination of *cdc25B* transcripts occurs by the end of the MZT in vertebrate and invertebrate species [32, 33]. Other maternal mitotic regulators included *cyclins* and *retinoblastoma-associated protein*, one of the key factors that controls the entry into cell cycle. Genes encoding the *mitotic checkpoint serine/threonine kinase bub1* ensuring correct chromosomal segregation during the cell division and the *wee1-like protein kinase* regulating DNA replication prior to mitosis were expressed until MBT and are likely to be involved in securing the integrity of the genome prior to cell division [34]. Several cell cycle related genes were found at 2hpf, including *centromere protein J,* which participates in centriole duplication [35], and the positive cell cycle regulator *smad nuclear interacting protein* [36]. The negative regulator, *cyclin-dependent kinase inhibitor 1C* was induced at MBT in concordance with reduction of cell proliferation.

Early fish embryos possess a multifaceted defence system. A suite of immune genes was expressed at high levels already in UFO. This group included *complement components*, *cytokines*, *chemokines* and their *receptors*, *IFN* and *TNF*-related genes, together with three negative regulators of immunity from the *SOCS* family. Several immune genes including *myeloperoxidase* were activated during SGM. While protection from DNA damage was driven mainly by the maternal transcripts, responses to oxidative stress were switched on later, and seven of eight genes involved in regulation of redox homeostasis were induced during SGM (Figure 2C). Marked developmental regulation was shown by *glutathione peroxidases* and *oxidation resistance protein* coding genes.

### Cell-to-cell and cell-to-extracellular matrix (ECM) interactions

**Figure 3:**
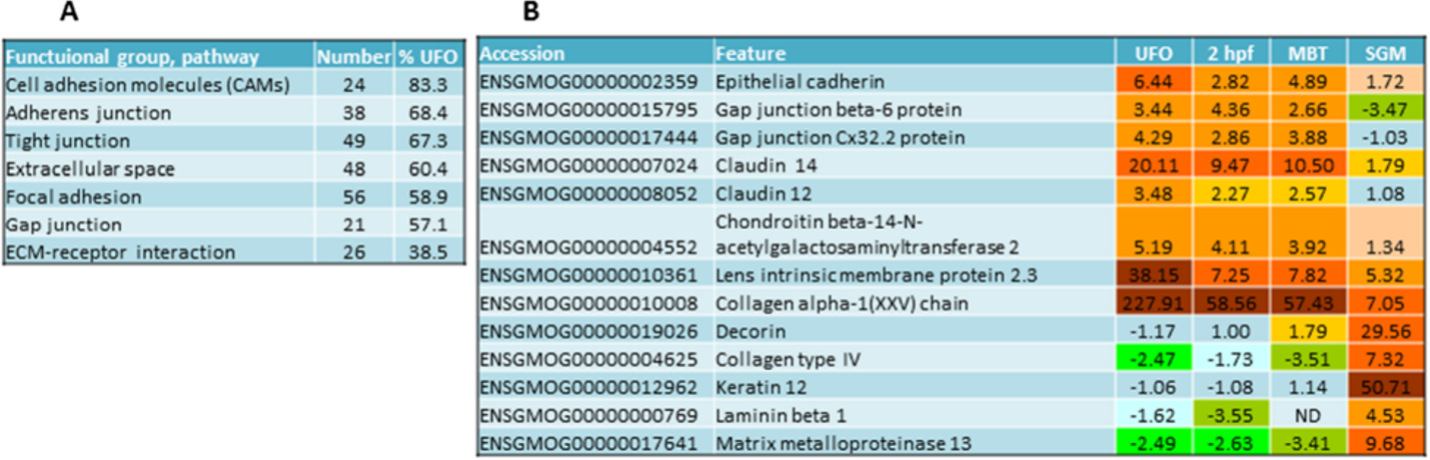
**Cell-to-cell interactions and ECM. A**: numbers of genes by functional groups and pathways involved in regulation of communication and contacts between cells. **B**: expression patterns of genes coding for proteins involved in cell adhesion and cell-to-ECM interactions.

The maternal transcripts were predominant among cell adhesion molecules and their input was high in other functional groups and pathways implicated in cell contacts and interactions (Figure 3A). Abundance of transcripts for *cadherins* (11 genes), *claudins-12* and *14, and gap junction proteins* was high in UFO and decreased sharply at SGM (Figure 3B). This stage was also marked with massive rearrangement of the extracellular space. Expression of two genes encoding the extracellular proteins, *lens intrinsic membrane protein 2.3* and *collagen XXV,* was highly specific for oocytes, and the latter showed 227-fold greater abundance in comparison with adult tissues and remained highly abundant until SGM (Figure 3B). These proteins are scarcely explored and it is unknown whether they are required for the oocyte maturation or embryonic development. Late activation of *decorin*, *laminin beta 1* and *collagen type IV* witnessed increased complexity and mechanical strength of the extracellular structures (Figure 3B). Up-regulation of collagen degrading *matrix metalloproteinase 13* was probably required for remodeling of extracellular matrix. High induction was shown by four genes for *keratin 12* that is commonly located in epithelium being involved in interactions between cells and ECM.

### Transcriptional repression/activation and chromosomal remodeling

**Figure 4:**
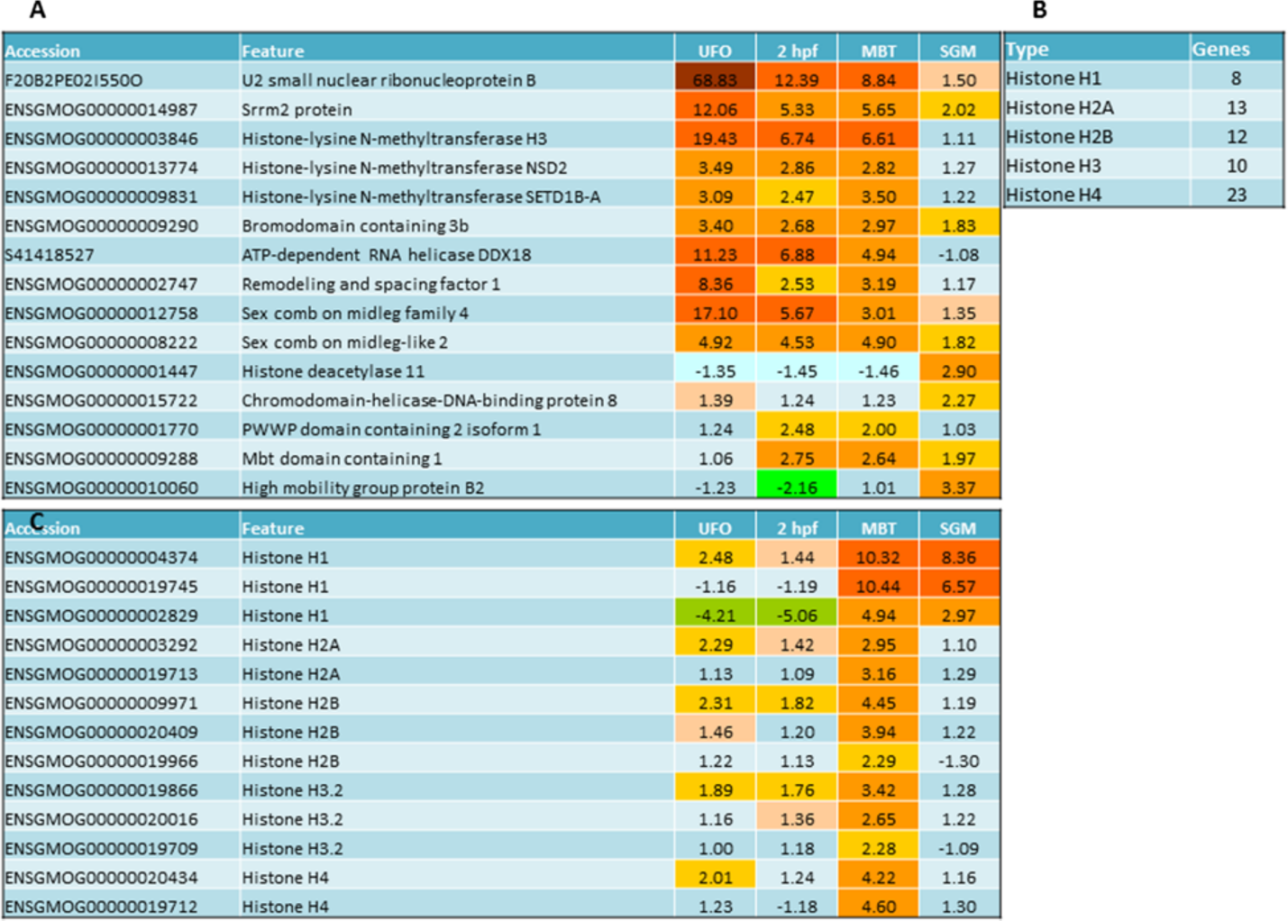
**Transcriptional regulation and chromosomal remodeling. A**: expression of genes with known functions in RNA processing, transcriptional regulation, chromosome maintenance and remodeling. **B**: number of activated genes from different histone classes. **C**: expression of transcripts coding for different histones.

The UFO transcriptome encoded proteins involved in mRNA processing, some of which showed highly specific expression in oocytes. For example *U2 small nuclear ribonucleoprotein B* displayed 69-fold higher levels compared to adult tissues (Figure 4A). The maternal transcripts of *RNA helicase DDX18* encode a protein taking part in spliceosome assembly suggesting that the regulation of mRNA splicing and processing continues even after arrest of transcription in oocytes and early embryos. Transcription repressors and activators seemed to be associated with male and female gametes. UFO included several *histone-lysine N-methyltransferases H3,* which have been implicated in transcriptional gene silencing, heterochromatin assembly and DNA methylation [37]. UFO transcripts also encoded components of the polycomb repressive complex contributing to the formation of silent chromatin. *Sex-comb on midleg-like proteins* are required to maintain the transcriptionally repressive state of homeotic genes throughout the development [38]. Further, the transcriptional repressors *remodeling and spacing factor 1* (*rsf1*) and *mbt domain containing 1* [39] were present in unfertilized and fertilized oocytes, respectively. A maternally supplied co-activator *bromodomain containing 2* (*brd2*) [40, 41] can be important for the early embryo cell cycle control. Fertilized oocytes contained transcripts for a protein involved in DNA methylation (*PWWP domain containing 2 isoform 1*). Though a number of genes related to chromosome maintenance and remodeling were present among prezygotic transcripts, major changes took place at MBT and a large fraction of the activated genes encoded histones (66 genes) (Figure 4B). Histone modifications and changes of chromatin architecture enable the formation of transcriptionally active euchromatin in order for zygotic gene expression to take place [20, 42]. Although some of the transcripts coding for histones H1, H2A, H2B, H3 and H4 were present at relatively high levels already in oocytes, most of them markedly increased abundance at MBT and many had biphasic profiles (Figure 4C). Several genes involved in nucleosome and chromatin remodeling, including *chromodomain helicase DNA binding protein 8*, *histone deacetylase 11* and *high mobility group protein b2* showed highest expression at SGM (Figure 4A).

### Regulation of early cellular differentiation and signaling

**Figure 5:**
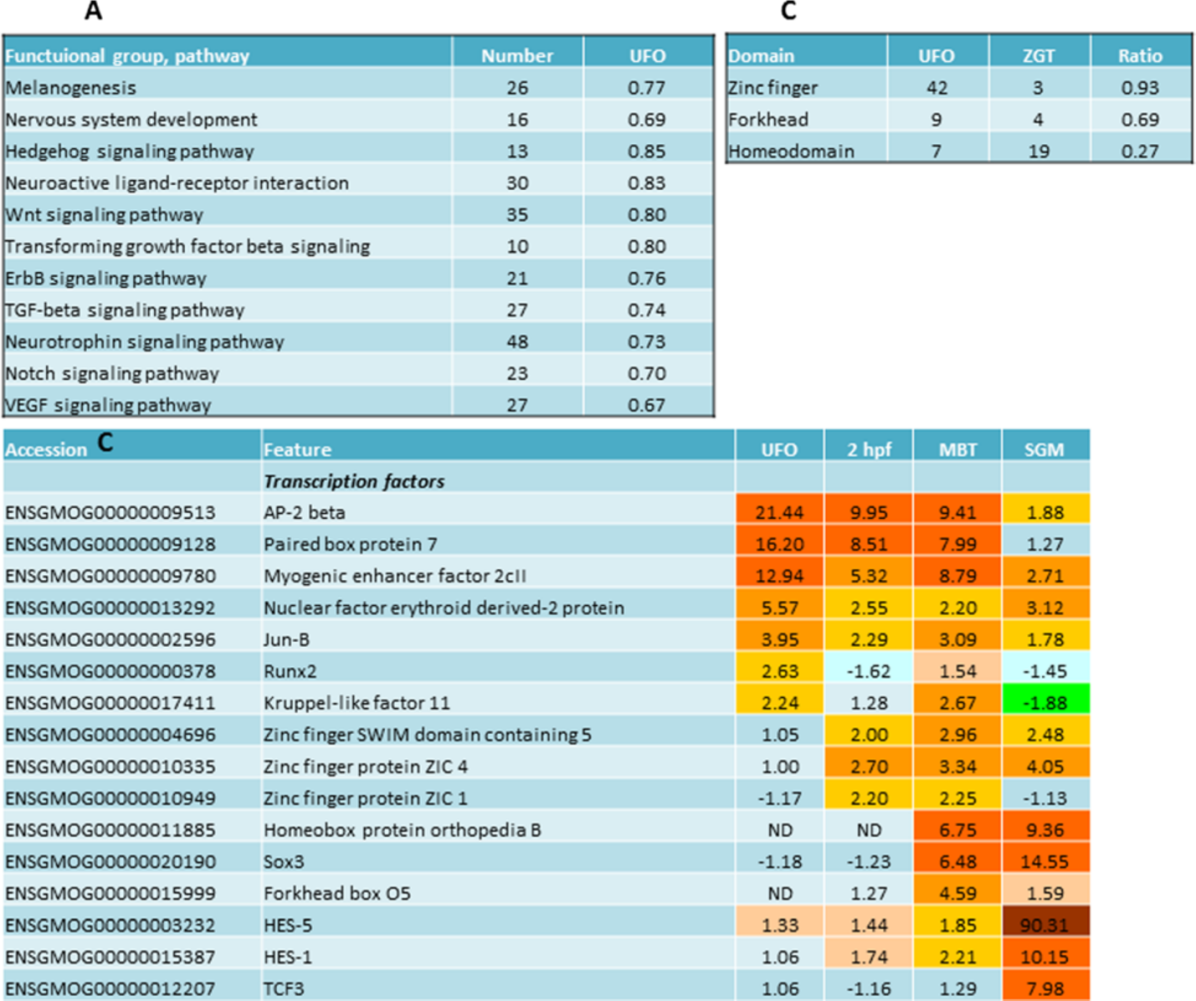
**Regulation of embryonic development. A**: numbers of genes by pathways with important roles in embryogenesis. **B**: expression patterns of various transcription factors controlling embryogenesis. **C**: developmental expression of transcription factors with different domains. Abbreviations are as follows: PZGT- prezygotic, ZGT-zygotic.

Many developmentally regulated genes belong to pathways with crucial roles in embryonic development (Figure 5A). A large part of these genes are transcription factors with different temporal expression profiles (Figure 5B). Transcripts for *jun-B* that controls differentiation of diverse cell lineages [43, 44], *pair box protein 7* and *AP-2* involved in formation of eye, limb, neural and cardiac development [45] were highly abundant in oocytes. UFO also included transcripts for proteins that play important parts in development of heart (*myogenic enhancer factor* [46]), cartilage and bone (*runx2*) and erythroid lineage (*nuclear factor erythroid derived-2*). Four transcription factors detected at 2hpf contained zinc finger domains. Greatest induction in SGM (90.3-fold) was shown by *hes-5*, a component of Notch pathway [47] and several homeodomain genes. Many identified transcription factors are unknown but include functional domains present in multiple regulators of development and interestingly, ratios between regulators with domains changed by stages (Figure 5C). While the majority of *zinc finger proteins* were found among prezygotic transcripts, 73% of transcription factors with *homeodomain* were activated during SGM, while *forkhead proteins* occupied an intermediate position.

**Figure 6:**
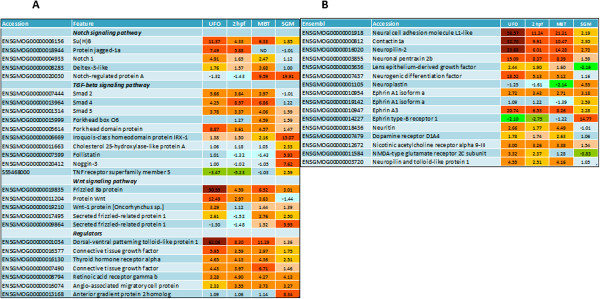
**Expression of genes involved in regulation of embryonic development. A**: receptors and extracellular proteins. **B**: regulators of neural system development.

In addition to transcription factors, the developmentally regulated genes included transcripts for receptors and extracellular proteins assigned to Notch, TGF beta and Wnt signaling pathway (Figure 6A). Major part of them were present in oocytes and only two genes were each activated at MBT and SGM. Greatest differences from adult tissues (51- and 62-fold) were observed in *frizzled 8a*, a receptor for Wnt proteins, and *dorsal-ventral patterning tolloid-like protein*. Dorsoventral patterning is also regulated by two genes from TGF pathway: *follistatin* and *noggin* [48]. Maternal transcripts encoded three proteins from Smad family, which transmit signals from TGF. *Notch* are transmembrane proteins that bind *jagged* ligands controlling differentiation by receiving signals through cell to cell contacts, while *deltex* is a regulator of notch signaling. *Su(H)B* encodes the *suppressor of hairless*, a key transcriptional regulator of Notch pathway. Of note is oocyte expression of genes that regulated differentiation of complex structures, such as *connective tissue growth factor* (3 genes) and *angio-associated migratory cell protein*.

A suite of genes known for their roles in neurogenesis was detected already in UFO (Figure 6B). Greatest difference with adult tissues (59-, 34- and 33-fold) was shown by *neural cell adhesion molecule L1-like protein (chl1)* implicated in cell migration and neuronal positioning, *neuropilin* and *contactin-1a,* a neuronal cell adhesion molecule important for the formation of axon connections during the nervous system development [49, 50]. Maternally provided transcripts also included *neurogenic differentiation factors* (5 genes)*,* which are involved in neuroepithelial stem cell differentiation and neurogenesis, the synaptic protein and receptor of neurotransmitters *neuronal pentraxin-1 precursor (nptx1)* and two *ephrins* and *ephrin type-B receptors* (4 genes), that play a crucial part in migration of axons. Interestingly, switch of *ephrin* and *ephrin receptor* isoforms took place at SGM. Maternal transcripts also encoded receptors of the dopamine and acetylcholine neurotransmitters.

### Master regulators of embryogenesis

**Figure 7:**
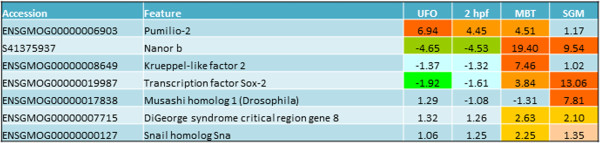
**Expression of selected master regulators of development.**

The microarray analyses revealed several genes that control stem cell fates and organ development, which were active at different stages (Figure 7). While transcript for *pumilio-2* (proliferation and renewal of stem cells, germ cell development and degradation of maternal mRNAs [51]) was present in oocytes, *snail homolog sna* (formation and maintenance of mesoderm during embryogenesis [52]), RNA-binding protein *musashi homolog 2* (proliferation and maintenance of stem cells in the CNS [53]) were activated at MBT and SGM, respectively. Similar to zebrafish [54], MBT was marked with an onset of *nanor* transcription. *DiGeorge syndrome critical region gene 8* may be involved in the degradation of maternal transcripts via biogenesis of microRNAs [55]. A role of microRNAs, such as miR430, in the clearance of maternal transcripts during the maternal to zygotic transition has been well documented [56, 57]. *Sox-2* (3 genes) is a key regulator of embryonic stem cell pluripotency [58].

### Validation of microarray data

**Figure 8:**
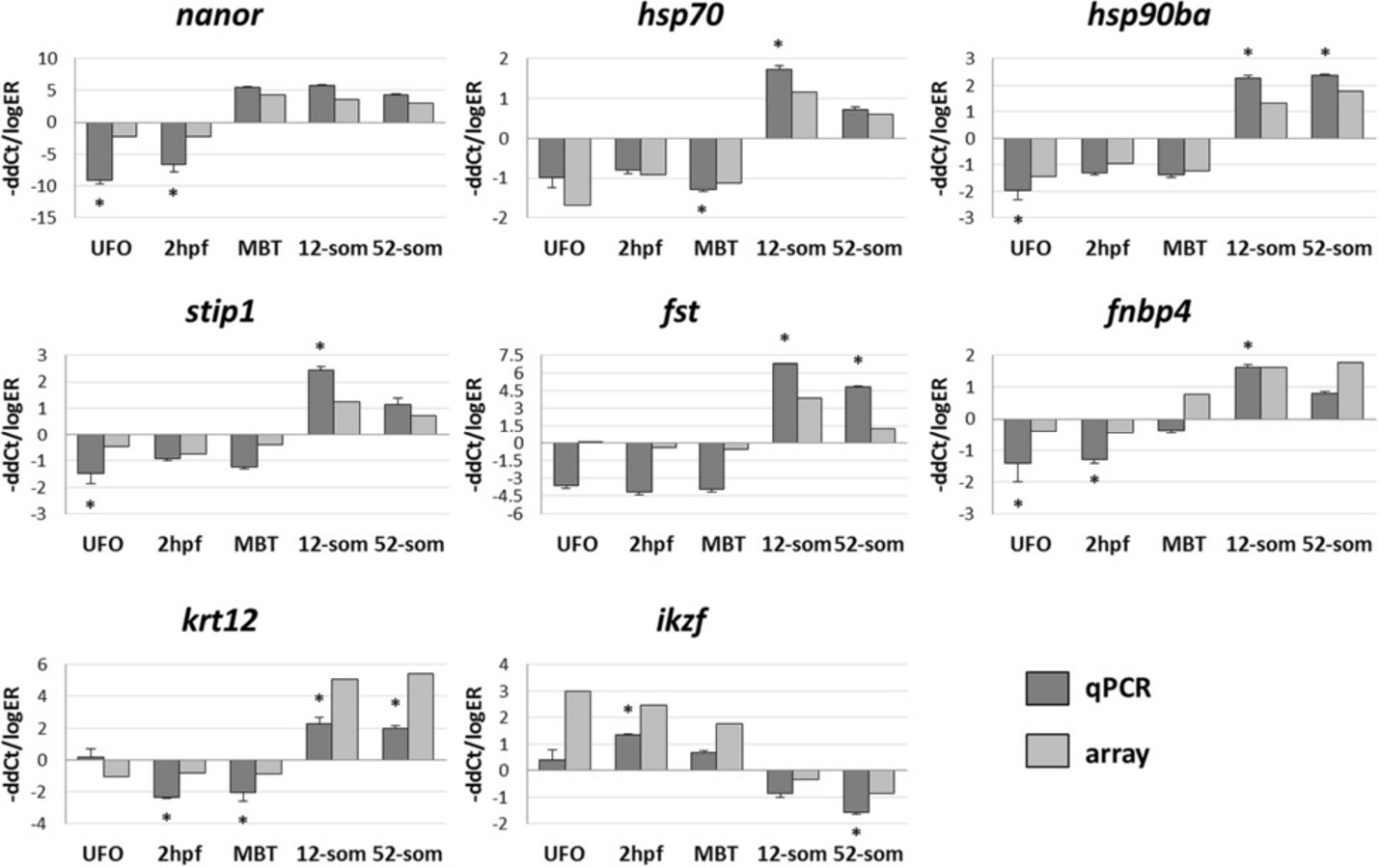
**Quantitative real-time PCR validation of the selected eight genes identified in the microarray analysis.** Data are presented as –ΔΔCt ± SE for qPCR and log_2_ expression ratio (log_2_ –ER) for microarray. Six pools of ten oocytes/embryos were used for the analyses (n=6). Significant difference detected by qPCR is marked with *, p < 0.05.

To validate microarray data, real-time qPCR was performed on eight genes: *nanor*, *heat shock protein 70 kDa protein 4* (*hsp70*), *heat shock 90 kDa protein 1 beta isoform a* (*hsp90ba*), *stress-induced phosphoprotein 1* (*stip1*), *follistatin* (*fst*), *formin-binding protein 4* (*fnbp4*), *keratin-12* (*krt12*) and *ikaros* (*ikzf*) at the embryonic stages of UFO, 2hpf, MBT, 12- and 52-somites. Overall, microarrays and qPCR produced similar results (Figure 8).

## Discussion

Genome sequencing enabled construction of oligonucleotide microarrays that may provide complete coverage of the polyadenylated fraction of transcriptomes; microarray analyses evaluate abundance of mature mRNA, which is capable for translation. While a genome-wide platform was used for evaluating the abundance of mature mRNA during zebrafish development [10], we report the first study performed with an aquaculture fish species. The main issue was the presentation of pathways and functional groups among the transcripts displayinh different temporal profiles. High complexity of the transcriptome in unfertilized cod oocytes is consistent with similar studies in both invertebrates and vertebrates [10, 59, 60]. Maternally provided mRNA comprised the major part of prezygotic transcripts while the putative paternal contribution was small, but sperm transcripts might play important roles of in the establishing of early embryonic gene expression profiles [5, 61, 62]. Fertilized cod eggs contained a suite of transcripts for proteins involved in chromatin remodeling and regulation of transcription, cell cycle control and cellular transport. However, it is unknown whether these genes have any developmental roles. In general, maternal transcripts support basic requirements of the embryo prior to the onset of zygotic expression. Interestingly, we got evidence that processing of mRNA continues even in absence of transcription that is in line with recent report on large-scale maturation of maternal transcripts in zebrafish embryos [57, 63]. In addition to maintenance of metabolism, cell structure and proliferation, transcripts of oocytes provide immune protection against pathogens and a suite of genes is expressed at higher level in comparison with adult tissues. Maternal transfer of complement factors and their protective roles was reported in wolffish, rainbow trout and zebrafish [64–67]. The female fish also provide offspring with immunoglobulins, lysozymes, protease inhibitors and different types of lectins [68, 69]. The observed prevalence of immune genes involved in signaling suggests that embryos are capable to regulate responses to pathogens. Presentation of multiple signal transduction pathways points to active perception of external cues and complex interactions between early embryos and environment.

Differentiation presumes acquisition of specific properties by cells and increase of their heterogeneity. A large number of transcripts for proteins involved in cell contacts were abundant in UFO being eliminated at SGM. Cadherins are transmembrane cell adhesion proteins that mediate various processes during development including cellular migration and tissue organization [70]. Interestingly, this study identified a large number of cadherin paralogs that are likely involved in cell sorting and tissue morphogenesis [71]. UFO included many transcripts that can be involved in the control of processes taking place long after fertilization, such as components of Wnt, Notch, hedgehog, ErbB, TGF beta and VEGF signaling pathways and markers of specialized cell lines [72, 73]. We also identified multiple transcripts that may regulate neurogenesis or encode proteins known as highly specific for neural tissue, in agreement with a few studies reporting maternal deposition of transcripts later expressed in the CNS (e.g. *Drosophila*, zebrafish and axolotl [74–76]). This finding can partly be accounted for by the bias in annotation, since a number of genes with pleiotropic functions have been studied mainly in the context of nervous system. Furthermore, some genes could change functions in course of the vertebrate evolution as demonstrated by the identification several genes known as neural specific in mammals were primarily involved in innate antiviral responses in fish [77, 78].

Transcriptome analyses suggested that the onset of zygotic expression is preceded by large scale modification of chromosomes. Histones comprise a major fraction of genes activated during MBT while the number of transcription factors in this group was small. The pre-MBT transcripts encoded several proteins that modify histones and DNA and are known as positive and negative regulators (e.g. *myst2*, *brd2*, *n6amt1*, *rsf1*, *ehmt3*, *scml-1*, *2* and *4*). Transcripts coding for histone methyltransferases and members of the polycomb repressors were highly abundant in unfertilized and fertilized oocytes, but showed a decrease in expression after MBT and coincided with the chromatin remodeling prior to the activation of transcription. Preparation of transcriptional machinery to the large-scale activation of gene expression appears a major developmental event that takes place during MBT. In most studied vertebrates this period coincides with the degradation of maternal transcripts and activation of the zygotic genome which takes over the genetic control of embryogenesis [10, 79]. Furthermore, accumulated studies reveal the dynamic nature of chromatin regulation and the importance of its modifications during transitions from maternal to zygotic control of development [20, 42, 80]. Our data are consistent with recent studies reporting the activation of zygotic transcription at MBT in Atlantic cod [30, 81]. As large fraction of genome is transcriptionally inactive, rearrangement of chromatin is essential to provide an access of transcription factors to the *cis*-regulatory elements [82]. Microarray analyses are insufficient for accurate timing of the onset of transcription. Part of transcripts appear in the polyadenylated fraction due to maturation of maternal RNA [57, 63]. However, given modification of chromosomes during MBT and the size of the the SGM group, it is likely that a large part of mRNA denoted as zygotic was indeed transcribed from the zygotic genome. The SGM group was complex by composition and contained numerous developmental regulators. Massive upregulation of homeobox genes at SGM is consistent with their involvement in the establishment of body plan and formation of anterior-posterior axis of the embryo [83, 84]. Homeobox transcription factors and cell signaling pathways cooperate to pattern tissues and organs and to specify the fate of a variety of cell types. However, none of the functional groups and pathways was restricted to the post-MBT period and all were largely represented among UFO.

## Conclusion

Transcriptome profiling of the oocytes and embryos of Atlantic cod with an aid of genome-wide microarray provided an insight in events taking place in early development and the roles of parental and zygotic transcripts. Maternal transcripts are involved in cellular metabolism, signal perception and transduction, defence, communication and contacts between cells. High representation of pathways and genes that control development suggest early cell fate specification and patterning of tissues and organs, especially of the neuronal lineage. The key event of zygotic genome activation at MBT was extensive chromatin rearrangements followed by expression of multiple developmental regulators.

## Methods

### Ethical approval

The study was approved by the Norwegian Animal Research Authority and conducted according to the prevailing animal welfare regulations: FOR-1996-01-15-23 (Norway), European Convention for the Protection of Vertebrate Animals used for Experimental and Other Scientific Purposes (Strasbourg, 18.III.1986) and COUNCIL DIRECTIVE of 24 November 1986 on the approximation of laws, regulations and administrative provisions of the Member States regarding the protection of animals used for experimental and other scientific purposes (86/609/EEC).

### Sample collection

Atlantic cod eggs and embryos were obtained from farmed fish at the National Cod Breeding Centre (Kraknes, Tromsø, Norway). Eggs were hand stripped, fertilized *in vitro* and transferred to seawater rearing tanks at an average temperature of 4.5°C and 100% oxygen saturation. The following stages were selected for analyses: 1) unfertilized (UFO) and 2) newly fertilized oocytes, 2 hpf and embryos at 3) mid-blastula (MBT), 4) 12 somites and 5) 52 somites (end of somitogenesis). Embryonic stages were determined based on description of Atlantic cod development with precise timing [85]. Tissues from adult male and female cod were used as a reference in the microarray analyses. Eggs and tissue samples were stored in RNAlater (Ambion, Austin, Texas, USA).

### RNA extraction

Total RNA was extracted from Atlantic cod eggs and tissues using TRIzol (Life Technologies) and PureLinkTM RNA mini kit (Ambion, Austin, Texas, USA). For each developmental stage, 10 oocytes/embryos were pooled for the analyses. On-column DNase treatment was performed using PureLinkTM DNase (Life Technologies) in order to remove traces of DNA and impurities. The concentration was analyzed by NanoDrop ND-1000 spectrophotometer (Thermo Fisher Scientific, Wilmington, USA). The total RNA quality was assessed with Agilent 2100 Bioanalyzer (Agilent 2100 Bioanalyzer, Agilent Technologies, Waldbronn, Germany) and only the samples of high quality (RIN ≥ 8) were selected for analysis.

### Microarray analyses

The Nofima’s Atlantic cod oligonucleotide microarray (ACIQ-2) produced by Agilent Technologies in the 4 × 44 k format included 60-mer probes to the unique transcripts from Ensembl and Unigene which were annotated by functional categories of GO and pathways of KEGG using bioinformatics package STARS [78, 86]. The genes were assigned to the orthology groups of OrthoDB [87]. Three and two biological replicates of the respectively three first and two last stages were analyzed in a total of 13 microarrays. Reference RNA was prepared by pooling equal amounts of RNA from pyloric caeca, liver, muscle, brain and male and female gonad to identify genes with increased expression in oocytes and embryos or developmentally regulated genes. The common reference design also made possible comparison between stages and finding of stage-specific genes. RNA amplification, labeling and fragmentation were performed using Two-Colour Quick Amp Labeling Kit and Gene Expression Hybridization kit following the manufacturer's instructions (Agilent Technologies). The input of total RNA used in each reaction was 100 ng. Individual samples were compared to the common reference; assignment of fluorescent labels (Cy5 and Cy3) was changed in each hybridization performed at 65°C at the rotation speed of 10 rpm for 17 hours in the oven (Agilent Technologies). The slides were washed with Gene Expression Wash Buffers 1 and 2 as described by the manufacturer and scanning was performed at 5 μm resolution using a GenePix Personal 4100A scanner (Molecular Devices, Sunnyvale, CA, USA). The laser power was manually adjusted and the “auto PMT” was enabled to adjust PMT for each channel such that less than 0.1% of features were saturated and that the mean intensity ratio of the Cy3 and Cy5 signals was close to one. Nofima’s bioinformatic package STARS was used for data processing and mining. After filtration of low quality spots flagged by FE, lowess normalization of log_2_-expression ratios (ER) was performed. Results for the two last developmental stages were highly similar and these samples were therefore merged and denoted as SGM (segmentation). Features that passed quality control in all samples of at least one stage and showed over 2-fold difference from reference were selected (Additional file 1). Further, the features were assigned to groups with different temporal profiles (Figure 1) according to criteria presented in Table 1 with minor manual editing. Data were submitted to GEO Omnibus (GSE58392).

### Quantitative real-time RT-PCR

Eight genes were selected for qPCR analyses based on the results of microarray analyses (Table 2). Gene expression measured by real-time qPCR was performed on the same samples that were used for microarray analysis. Primers were designed with Primer3 software and synthesized by Life Technologies. The amplicon lengths were set to be between 60 and 200 base pairs. The cDNA synthesis was performed on 1.5 μg total RNA using the SuperScript® VILO™ cDNA Synthesis Kit (Life Technologies) in a 20 μl reaction system according to the manufacturer's protocol. The specificity of PCR amplification was confirmed with melting curve analysis. Efficiency was checked from tenfold serial dilutions of cDNA for each primer pair. A 2 × SYBR® Green PCR Mastermix (Roche Diagnostics, Mannheim, Germany), 0.8 mM of each primer, and 4 μl of 1:10 diluted cDNA template were mixed in 12 μl reaction volumes. PCR was performed in duplicates in 96-well optical plates on Light Cycler 480 (Roche Diagnostics, Mannheim, Germany) under the following conditions: 95°C for 5 min (pre-incubation), 95°C for 5 s, 60°C for 15 s, 72°C for 15 s (amplification), followed by 95°C for 5 s and 65°C for 1 min (melting curve). 45 amplification cycles were performed. We tested some of the commonly used reference genes and they all showed substantial variation in expression levels between fertilized oocytes and somitogenesis in agreement with other embryonic studies of Atlantic cod [88]. Hence, we selected six other candidate genes that showed stable expression on microarray throughout the development and tested them as reference genes. Finally, the combination of five most stable genes (Table 2) was used for normalization and the data are given as –ddCt values.

**Table 2:** **Primer list for real-time qPCR**

Gene name	Gene symbol	Sequence (5′- 3′)	Product length, bp
*nanor*	*nanor*	ATCCAATACCCAACGGTTCA	92
		GCGATGAAATGGCTGAATCT	
*Heat shock 70 kDa protein 4*	*hsp70*	TGAACAGCGCTATGAACCAG	117
		TCATGATGGGGTTACAAGCA	
*Heat shock 90 kDa beta*	*hsp90ba*	CGAGGAGCACTACAACGACA	181
		GTCCTGCTTCTCCTTCATGC	
*Stress-induced-phosphoprotein 1 (Hsp70/Hsp90-organizing)*	*stip1*	CCGATGTCCTGAAGAGGTGT	141
		TCATGGCTAAGGGGTAGTCG	
*Follistatin*	*fst*	ACCTGGAAAGGACCAGTGTG	117
		GCACTTTCCCTGGTACTGGA	
*Formin-binding protein 4*	*fnbp4*	GCCTGACCTCCACAGATGTT	100
		CAACACGGACATCTTCATCG	
*Keratin 12*	*krt12*	GCCAAGACTGACCTGACCAT	188
		GCCTCGTAGTGTTCCCTGAC	
*Ikaros*	*ikzf*	ATGATCTCCGGGTCTGTGAG	165
		ACACTTGAGCTTTCCGTCGT	
**Reference genes**			
*ATP synthase subunit s mitochondrial*	*ATP5s*	AACAGGGTGGACTATGAGAGGA	114
		GTGATGCCAGCGTTCAAA	
*Eukaryotic translation initiation factor 3 subunit 3 gamma 40 kDa isoform CRA b*	*eIF3*	AGGACGACGCAGACTTTGAC	121
		ACGAAGGAGCCGTAGAAGGT	
*Tetratricopeptide repeat protein 39C*	*tpr39*	GAAACGGGCTGAGAGACTGA	63
		ATGACACCCAGGAAGCAGAG	
*Dehydrogenase/reductase SDR family member 11*	*dhrs11*	GGAGACAGAGTTTGCGTTCC	128
		GAGGGGCACTGAGGACATAA	
*Ubiquitin*	*ubi*	GGCCGCAAAGATGCAGAT	69
		CTGGGCTCGACCTCAAGAGT	

## Electronic supplementary material

Additional file 1: **Data are log2-Expression Ratio to reference.** Column S - groups (prezygotic and zygotic), column T - subgroups defined by the temporal profiles. (XLSX 985 KB)
